# Effect of bisoprolol on central aortic systolic pressure in Chinese hypertensive patients after the initial dose and long-term treatment

**DOI:** 10.17305/bjbms.2021.6483

**Published:** 2021-10-29

**Authors:** Weiwei Zeng, Brian Tomlinson

**Affiliations:** 1 Department of Pharmacy, Shenzhen Baoan Women’s and Children’s Hospital, Jinan University, Shenzhen, Guangdong Province, China; 2 Faculty of Medicine, Macau University of Science and Technology, Macau, China; 3 Department of Medicine and Therapeutics, The Chinese University of Hong Kong, Prince of Wales Hospital, Shatin, Hong Kong

**Keywords:** Bisoprolol, BPro^®^ device, central aortic systolic pressure, radial tonometry

## Abstract

We conducted a prospective open-label cohort study with the aim of examining the effects of the highly β1-selective agent bisoprolol on central aortic systolic pressure (CASP) after the first dose and after 6 weeks’ treatment and whether the CASP response could be predicted from the early response. Chinese patients with primary hypertension (BP ≥140/90 mmHg) on no therapy or background amlodipine were treated with bisoprolol 2.5 mg daily for 6 weeks. Brachial systolic BP (Br-SBP), resting heart rate (HR), and CASP were determined at baseline, 24 hours after the first dose, and pre-dose after treatment for 6 weeks using the BPro® device. In 42 patients (age 54 ± 9 years), the mean reductions in CASP and Br-SBP after 6 weeks of treatment were not significantly different from each other at −14.5 ± 12.7 and −15.4 ± 12.9 mmHg (both *p* < 0.01), respectively. Changes in CASP and Br-SBP were highly correlated after the first dose (r = 0.964, *p* < 0.01) and after 6 weeks (r = 0.963, *p* < 0.01) and the reductions in CASP after 6 weeks were also associated with the reduction in CASP after the first dose (r = 0.577, *p* < 0.01). Bisoprolol was shown to effectively reduce CASP and this effect was directly proportional to the reduction in Br-SBP and of a similar magnitude. More favorable CASP responses to long-term therapy may be predicted by greater reductions in CASP after the first dose.

## INTRODUCTION

Central aortic systolic pressure (CASP), the pressure exerted on the heart and brain and other organs, is thought to be more strongly related to vascular disease and future cardiovascular events compared with the corresponding brachial blood pressure (BP) [1-3], although in routine clinical practice, only brachial systolic BP (Br-SBP) and brachial diastolic BP (Br-DBP) levels are measured.

Moreover, studies such as the Conduit Artery Function Evaluation (CAFE) study have revealed that different drugs may have different effects on CASP and the central hemodynamics while having similar effects on Br-SBP [4]. Therefore, evaluation of the effect of antihypertensive drugs on CASP is thought to be important [1,3].

Studies have shown that central BP was superior to brachial BP in predicting cardiovascular risk and chronic kidney disease, especially in the elderly [5]. A population study in Taiwan with a 10-year follow-up has shown a stronger association of target organ damage and cardiovascular mortality with central systolic pressure compared to brachial SBP and PP in normotensive and untreated hypertensive subjects [6]. In other studies, CASP showed a stronger correlation than Br-BP with the left ventricular mass [7] and C-PP was more strongly correlated than Br-PP with carotid intima-media thickness [2].

Beta-adrenoceptor antagonists or β-blockers are considered to be an appropriate therapy for hypertensive patients with additional indications, such as heart failure or angina, but they are no longer recommended as a general first-line therapy in many guidelines because clinical trials found that they were less effective than other antihypertensive drugs for the prevention of stroke [8,9]. One of the reasons β-blockers may be less effective in preventing stroke is that reduction in CASP is less with β-blockers than with other groups of antihypertensive drugs, which may be related to slowing the heart rate (HR) and increased wave reflection [10]. Most antihypertensive drugs reduce CASP to a slightly smaller extent than Br-SBP, but the reduction in CASP appears to be less with β-blockers compared with other classes of antihypertensive drugs for the same reduction in Br-SBP [11].

A number of clinical studies have demonstrated that therapy based on atenolol, which is one of the most widely used β-blockers, was significantly less effective for lowering CASP and central pulse pressure (C-PP) than other therapies [4,12]. However, there are fewer studies on the effects on CASP with bisoprolol, which is a highly β_1_-selective β-blocker that may have advantages over less selective β-blockers in the treatment of hypertension, such as less adverse metabolic effects [13].

Many previous studies used the SphygmoCor^®^ device (AtCor Medical, Sydney, Australia) to detect the arterial waveform using applanation tonometry and to estimate CASP using a generalized transfer function. Another watch-like device, the BPro^®^ (HealthSTATS Int’ Pte Ltd., Singapore), has been developed to measure CASP using the N-point moving average method, a mathematical low-pass filter [14]. The BPro device is not only capable of 24 hours ambulatory BP monitoring (ABPM) but also for capturing radial arterial waveforms and this tonometric method has been validated against the gold standard of direct aortic root measurement during cardiac catheterization, with excellent correlation (r = 0.99) [14]. The BPro device was used in a recent study in Singapore to assess the effect of valsartan on CASP in Asian hypertensives [15].

The present study was performed to investigate the effect of bisoprolol on CASP after the first dose and after 6 weeks’ treatment in Chinese patients in Hong Kong with mild-to-moderate hypertension using the BPro device and to analyze the relationship between changes in brachial and central BP and whether the response to the first dose might predict the long-term response.

## MATERIALS AND METHODS

### Subjects and study design

The study was approved by the local Clinical Research Ethics Committee and was performed in accordance with the ethical standards laid down in the Declaration of Helsinki and subsequent revisions. All patients provided written informed consent. This was an open-label study in hypertensive patients who consented to participate. We recruited either treatment-naïve hypertensive individuals or subjects who had stopped or reduced antihypertensive medications for at least 2 weeks and had BP levels in the required range. At the baseline evaluation, the subjects were required to have Br-SBP >140 mmHg and <170 mmHg and/or Br-DBP >90 mmHg and <110 mmHg after resting in the sitting position for at least 5 minutes.

Subjects with secondary hypertension, unstable angina, history of myocardial infarction, stroke or coronary heart disease (coronary bypass or angioplasty) in the previous 3 months before recruitment, heart failure (New York Heart Association II-IV), hemodynamically relevant aortic or mitral valve disease, obstructive hypertensive cardiomyopathy, abnormal heart rhythm or resting HR <60 beats/minutes at baseline (before starting bisoprolol treatment), primary hyperaldosteronism, renal artery stenosis, impairment of hepatic or renal function as defined by liver function values of ALT ≥1.5-fold the upper normal limit or serum creatinine >150 μmol/L or on investigator decision, and history or intolerance or with a known contraindication to β-blockers were excluded from the study. Anthropometric measurements of body weight and height were recorded utilizing standard equipment and methodology.

Patients received a once-daily dose of bisoprolol 2.5 mg at baseline and this was continued for 6 weeks. After 5 minutes of resting seated, brachial BP was measured 4 times at 2 minutes intervals in the dominant arm with an automatic device (Omron HEM 7080IT, Omron Healthcare). The average of the last three measurements was used in the statistical analyses.

CASP and C-PP were derived by the BPro^®^ wrist ABPM device using the A-Pulse CASP^®^ Application Software program (HealthSTATS Int’ Pte Ltd., Singapore). The tonometer in the wrist strap of the BPro was placed over the radial artery to capture the radial pulse waveform. The brachial BP value was entered into the software and the BPro device and software estimate central aortic pressures from the radial pulse waveform. CASP, C-PP, Br-SBP, brachial pulse pressure (Br-PP), and HR were derived from the radial artery waveform obtained by pulse wave analysis using the BPro device at baseline, 24 hours after the first dose, and 24 hours after the dose after 6 weeks of treatment with bisoprolol 2.5 mg daily. The systolic BP augmentation (SBPA) was calculated as Br-SBP minus CASP and pulse pressure (PP) ratio as the ratio of Br-PP to C-PP.

### Statistical analysis

Continuous variables are presented as the mean ± SD. Categorical variables are reported as the number of individuals (percentage). Comparison of categorical variables was done using Chi-square test and comparison of continuous variables was done using the two sample t-test. Comparisons between visits were performed using paired t-tests in subjects who attended for both visits. Linear regression analysis was performed to determine significant independent predictors of reduction in CASP after 6 weeks’ treatment and changes in SBPA and PP ratio.

The normality assumption was tested using the Kolmogorov–Smirnov test. The statistical analysis was performed using SPSS software, version 19 (SPSS Inc., Chicago IL, USA).

## RESULTS

### Demographic characteristics of participants

**FIGURE 1:**
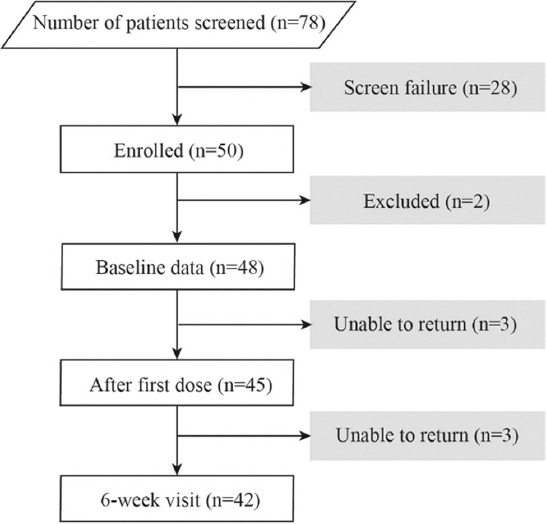
Flow diagram of the study

A total of 78 eligible patients were invited to participate in the study but 19 subjects were excluded during screening, mainly because the baseline ambulatory BP was <140/90 mmHg and nine patients withdrew because they did not want to perform ABPM. Two patients who had unusually high plasma bisoprolol concentrations, which were measured after 6 weeks’ treatment as part of a separate study, were excluded from the analysis. Three patients were unable to return for measurements after the first dose of treatment and another three patients did not return after 6 weeks so a total of 42 patients were included in this analysis. Baseline characteristics in 42 eligible patients with good adherence to bisoprolol treatment including 24 men and 18 women are shown in Table 1. The flow diagram of the study is shown in Figure 1.

**TABLE 1:**
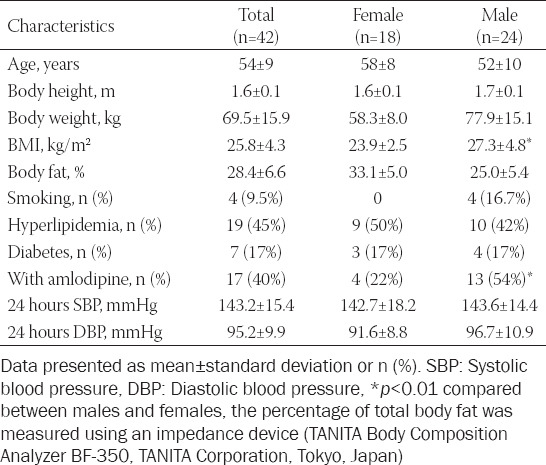
Baseline demographic characteristics of Chinese hypertensive patients (n=42)

From the 42 patients in the analysis, there were 17 (40%) with concomitant antihypertensive treatment with amlodipine. Nineteen (45%) patients diagnosed as having hyperlipidemia were taking lipid-lowering medication and 7 patients (17%) with type two diabetes mellitus were on glucose-lowering medication. There was a significantly greater proportion of male compared with female patients with concomitant amlodipine treatment (Table 1). The male patients tended to have lower C-PP compared with female patients and the PP ratio was significantly higher in male compared to female patients (1.25 ± 0.12 vs. 1.15 ± 0.06, *p* < 0.05). No other significant difference was observed between male and female patients. All the 42 patients completed ABPM and were confirmed to have uncontrolled hypertension at baseline and started treatment with bisoprolol. The baseline 24 hours SBP was 143.2 ± 15.4 mmHg and 24 hours DBP was 95.2 ± 9.9 mmHg.

### Effects of bisoprolol on central BP and brachial BP

At 24 hours after the first dose, there were significant reductions in Br-SBP, Br-DBP, Br-PP, HR, CASP, and C-PP (Table 2). There were small but significant reductions in SBPA and PP ratio. After 6 weeks of treatment, there were also significant reductions in Br-SBP, Br-DBP, Br-PP, HR, CASP, and C-PP but there were no significant changes in SBPA and PP ratio compared to baseline. The Br-SBP, Br-DP, and CASP were significantly lower at 6 weeks compared to after the first dose. The change in CASP after 6 weeks was not significantly different from the change in Br-SBP (*p* = 0.088).

**TABLE 2:**
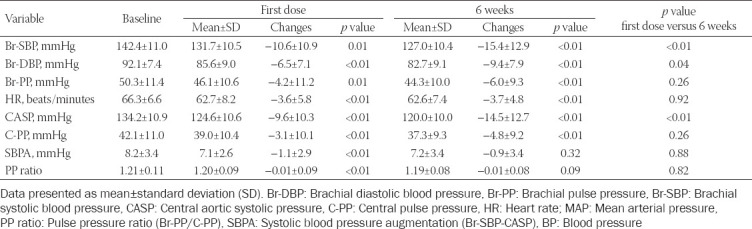
Changes in brachial and central BP parameters with bisoprolol treatment 24 hours after the first dose and pre-dose at 6 weeks (n=42)

Comparing the response to bisoprolol in male and female patients, the reduction in HR was significantly greater in females than males (−4.3 ± 3.8 vs. −3.1 ± 7.0 beats/minutes, *p* < 0.05) after the first dose but there was no significant difference after 6 weeks’ treatment. The reduction in PP ratio was significantly greater in males than females (−0.003 ± 0.057 vs. −0.018 ± 0.106, *p* < 0.05) after the first dose but not after 6 weeks’ treatment. There were no other significant differences in responses between male and female patients (Supplementary Table 1).

There were no significant differences in responses between patients with and without concomitant amlodipine treatment (Supplementary Table 2).

Univariate linear regression analysis showed that the factors significantly associated (*p* < 0.001) with the reduction in CASP after 6 weeks’ treatment were baseline Br-SBP, HR and CASP, the reduction in Br-SBP after 6 weeks’ treatment, the reduction in CASP after the first dose, and the HR after the first dose of bisoprolol (Table 3). Multivariate analysis showed that only baseline CASP and Br-SBP, and the reduction in Br-SBP values after 6 weeks’ treatment were significantly associated with the reduction in CASP after 6 weeks’ treatment.

**TABLE 3:**
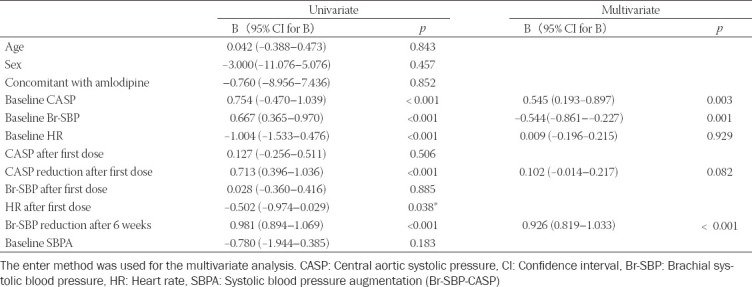
Linear regression analysis of CASP reduction after 6 weeks based on below variables

**FIGURE 2:**
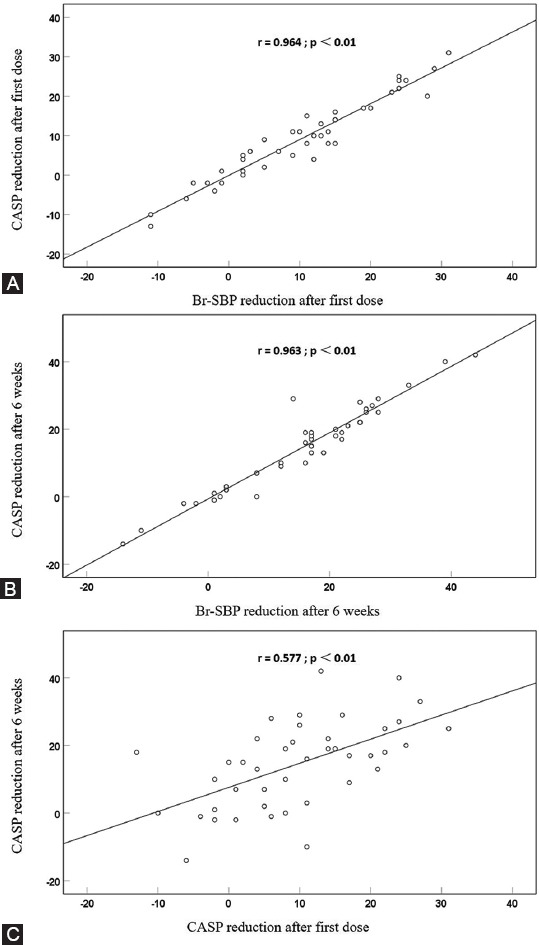
Scatter plots and lines of best fit showing the relationships for (2a) reductions in CASP and Br-SBP after first dose of bisoprolol; (2b) reductions in CASP and Br-SBP after 6 weeks’ treatment with bisoprolol; (2c) reductions in CASP after 6 weeks’ treatment with bisoprolol and after the first dose of bisoprolol.

Correlation analysis demonstrated strong correlations between the reductions in Br-SBP and CASP after the first dose (r = 0.964, *p* < 0.01) and after 6 weeks’ treatment (r = 0.963, *p* < 0.01) and between reductions in CASP after the first dose and after 6 weeks’ treatment (r = 0.577, *p* < 0.01, Figure 2). Linear regression showed that for every 1.0 mmHg reduction in Br-SBP, there was a corresponding 0.6 mmHg drop in CASP.

## DISCUSSION

The reductions in CASP and C-PP (−14.5 ± 12.7 mmHg and −4.8 ± 9.2 mmHg) after 6 weeks treatment with bisoprolol 2.5 mg in this study were very similar to the changes after 4-8 weeks treatment with bisoprolol 5 mg (−14 ± 10 mmHg and −3 ± 10 mmHg) reported in a study in Chinese hypertensive patients in Shanghai using the SphygmoCor device [16]. That study compared the responses to bisoprolol 5 mg and atenolol 50 mg and with atenolol, the changes in CASP and C-PP were significantly different to those with bisoprolol at −6 ± 9 mmHg and +3 ± 8 mmHg, respectively, despite similar reductions in Br-SBP, Br-PP, and HR with atenolol and bisoprolol. The reduction in PP amplification ratio was also greater with atenolol than bisoprolol.

However, two other studies have shown that the effects on central hemodynamic of bisoprolol and atenolol effects similar. A study in Korea using the SphygmoCor device randomized 209 hypertensive patients, who were on no other treatment, to treatment with bisoprolol 5 mg or atenolol 50 mg once daily for 12 weeks with a possible titration to double these doses if BP was not controlled at the 4^th^ week [17]. The mean reductions in brachial SBP and aortic SBP were not significantly different with bisoprolol and atenolol at 19.10 mmHg and 15.35 mmHg, respectively, with bisoprolol and 17.63 mmHg and 12.71 mmHg, respectively, with atenolol. Central PP was also significantly reduced to the same extent by both treatments, suggesting that both β-blockers may have a similar effect on central hemodynamics.

In contrast, in a study from Argentina involving 19 hypertensive patients with six on various background therapies, the patients were randomized to titrated doses of bisoprolol (2.5-5 mg) or atenolol (25-50 mg) each for 4 weeks in a crossover study using the SphygmoCor device to estimate central pressures [18]. Brachial SBP was significantly reduced by 10.63 ± 13.3 and 7.15 ± 11.3 mmHg after atenolol and bisoprolol, respectively, but the reductions in central SBP of 7.11 ± 13.1 and 4.05 ± 10.7 mmHg after atenolol and bisoprolol, respectively, were not significant. There was no change in central PP or brachial PP as diastolic pressures were reduced to a similar extent as systolic pressures. There may be various reasons why the patients in that study responded differently to those in the other studies. Five of the 19 patients were on treatment with an angiotensin-converting enzyme inhibitor or an angiotensin receptor blocker which may blunt the antihypertensive response to β-blockers.

In the present study, the reductions in CASP and C-PP with bisoprolol were similar to the changes (−15.3 ± 10.9 mmHg and −4.3 ± 10.1 mmHg) reported with valsartan at a dose of 80 mg daily or more for 12 weeks in a study using the BPro^®^ device in Asian hypertensive patients in Singapore [15]. In that study, there was a non-significant increase in the PP ratio and for every 1.0 mmHg reduction in brachial SBP, the reduction in CASP was 0.8 mmHg compared to 0.6 mmHg in the present study.

In the CAFE study, compared to the atenolol-based regimen, the amlodipine-based regimen produced significantly greater reductions in CASP (difference 4.3 mmHg; 95% CI, 3.3-5.4) and C-PP (difference 3.0 mm Hg; 95% CI, 2.1-3.9) and the pulse pressure amplification ratio was significantly higher with the amlodipine-based regimen (1.31; 95% CI, 1.3-1.32 vs. 1.21: 95% CI 1.2-1.21) but HR was significantly lower (difference −10.7 BPM; 95% CI −11.5-−9.8) with the atenolol-based regimen [4].

An analysis from the CAFE study found that there was a highly significant inverse relationship between HR and both CASP and C-PP, indicative of increased wave reflection at lower HR levels [7]. HR and Br-BP accounted for 92% of the variability in central systolic and pulse pressures. The authors concluded that HR reduction with β-blockers is a major mechanism accounting for less effective CASP reduction per unit change in Br-SBP.

In a study using a propensity score analysis, hypertensive participants using β-blockers had significantly greater C-PP (46.5 ± 12.9 mmHg) than matched nonusers (45.4 ± 11.0 mmHg) and the difference was greater without adjustment for HR, suggesting that the unfavorable central hemodynamic profile of β-blockers has both HR-dependent and HR-independent components, which were similar for all frequently used β_1_-selective β-blockers [19].

A study which examined the acute effects of atenolol on central BP made observations at least 3 hours after an oral dose of 50 or 100 mg atenolol and adjusted the HR by atrial pacing with a permanent pacemaker in patients with normal baseline BP [20]. The authors concluded that the inferior ability of atenolol to reduce central compared to peripheral BP can be explained by the combination of its HR-dependent and HR-independent effects. Among HR-independent mechanisms, they suggested slight vasoconstriction, resulting from unopposed α-receptor stimulation or β_2_-receptor blockade would bring reflection sites more proximal to the aorta and thus counteract CASP reduction.

In a meta-analysis of trials comparing the effects on CASP with vasodilating β-blockers and non-vasodilating β-blockers, non-vasodilating β-blockers, but not vasodilating β-blockers, resulted in a lower reduction in CASP than in Br-SBP, but the difference in treatment-induced SBP amplification changes was nearly abolished after accounting for differences in HR changes as the vasodilating β-blockers did not reduce HR to the same extent as non-vasodilating β-blockers [21].

In the present study, we evaluated the effects of bisoprolol on CASP in patients with mild-to-moderate primary hypertension. Bisoprolol was effective in lowering CASP and the reduction in CASP after 6 weeks’ treatment was not significantly different from the reduction in Br-SBP. These effects on Br-BP and central BP are consistent with data from the previous study in Chinese patients in Shanghai [16], but other studies have shown variable effects. It has been suggested that the lesser degree of reduction in central BP with β-blockers than with some other groups of antihypertensive is a major reason for their inferiority in prevention of stroke. Many of the trials used atenolol which may be less effective in reducing central BP than some other β-blockers and this may have contributed to β-blockers having less benefit in reducing stroke than other groups of antihypertensive drugs [22].

There are several factors that may affect central BP and the response to antihypertensive drugs and these may differ between studies. Concomitant diseases such as coronary heart disease (CHD) may be associated with increased central BP and patients with CHD were reported to have significantly higher CASP, central PP, and PP amplification than those without CHD [23]. Moreover, the cardiovascular risk factors such as diabetes, hypercholesterolemia, and smoking, which can accelerate stiffening in the large arteries, may also have greater effects on CASP than peripheral pressures and can contribute to differences between central and brachial BP [24-26].

Several antihypertensive drugs have shown different effects on central BP despite having the same influence on brachial BP because of their different ability to reduce central systolic BP and central pulse pressure. Many trials have found that angiotensin receptor blockers have favorable effects on central BP and arterial stiffness. In the Losartan Intervention for Endpoint Reduction in Hypertension study, total peripheral resistance index was reduced more with losartan-based therapy than atenolol-based therapy but the conduit artery stiffness assessed as pulse pressure/stroke index showed similar reductions with the two antihypertensive regimens, suggesting that they had comparable effects on arterial stiffness [27].

The present study has several limitations, the most obvious being there is no comparison of the effects of bisoprolol with any other treatment. The study was an open-label study so there may have been a placebo effect and the number of subjects is relatively small and somewhat heterogeneous. It would also have been useful to assess the responses to bisoprolol at the peak time after the dose and at other time points. We chose to make the measurements at 24 hours after the first dose so that it would be comparable to the measurements at 6 weeks which were made at 24 hours after the previous dose. BP responses are often measured at the trough level during long-term follow-up.

## CONCLUSION

This study demonstrated that a low dose of bisoprolol reduced CASP to a similar extent to the reduction in Br-SBP in Chinese patients with primary hypertension and this effect was similar to that previously described with valsartan. The reduction in Br-SBP at 6 weeks and greater reductions in CASP after the first dose predicted greater reductions in CASP after 6 weeks’ treatment and these measurements might be used to predict a good response in central hemodynamics with this dose of bisoprolol to help in individualizing treatment.

## Figures and Tables

**SUPPLEMENTARY TABLE 1:**
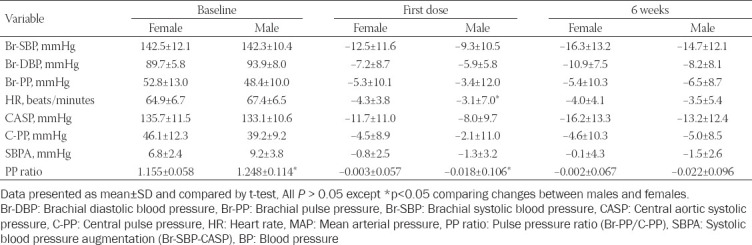
Changes in BP and central hemodynamics after the first dose and 6 weeks in male (n=24) and female (n=18) patients

**SUPPLEMENTARY TABLE 2:**
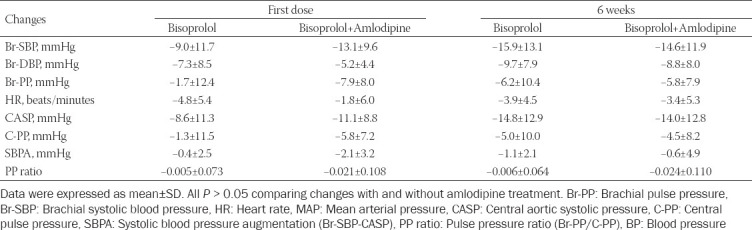
Comparison of changes in brachial BP and CASP after first dose and 6 weeks in patients on concomitant treatment with (n=17) and without amlodipine (n=25)
